# Ethnicity and anticoagulation management of hospitalized patients with atrial fibrillation in northwest China

**DOI:** 10.1038/srep45884

**Published:** 2017-04-10

**Authors:** Xinchun Cheng, Xianhui Zhou, Shifei Song, Min Wu, Roza Baolatejiang, Yanmei Lu, Yaodong Li, Wenhui Zhang, Wenkui Lv, Yuanzheng Ye, Qina Zhou, Hongli wang, Jianghua Zhang, Qiang Xing, Baopeng Tang

**Affiliations:** 1 Pacing and Electrophysiological Department, The First Affiliated Hospital of Xinjiang Medical University, Urumqi, Xinjiang 830011, China; 2 Cardre health care No. 4 department, The people’s hospital of Xinjiang Uygur Autonomous Region, Urumqi, Xinjiang, 830054, China; 3 The First Department of Cardiology, Hospital of Xinjiang Production & Construction Corps, Urumqi, Xinjiang, 830054, China

## Abstract

The therapeutic management and health challenges caused by atrial fibrillation (AF) differ between different groups. The purpose of this study was to investigate the clinical features of patients hospitalized with AF and to explore the use of anticoagulation treatments in Han and Uygur patients in Xinjiang, northwest China. Data were collected from a retrospective descriptive study involving patients hospitalized at 13 hospitals in Xinjiang, China from Jul 1, 2014 to Jun 31, 2015. Anticoagulation management was measured according to guideline-recommended risk scores. A total of 4,181 patients with AF were included (mean age 69.5 ± 11.7 years, 41.4% females; 71.5% Han, 28.5% Uygur). The prevalence of AF in Uygur individuals may occur earlier than in Han individuals (mean age 64.9 vs 71.3, P < 0.001). Most of the hospitalized patients with AF had a high risk of stroke (CHA_2_DS_2_-VASc score ≥2; 80.6% Han vs 73.7% Uygur, P < 0.05); this risk was especially high in elderly patients. In AF patients, the application of anticoagulants according to the guidelines is far from expected, and the underutilization of anticoagulants exists in both ethnic groups.

Atrial fibrillation (AF) is present in approximately 2.3 to 3.4% of patients and contributes to a progressively increasing and substantial healthcare costs^1^. AF-associated complications including stroke and vascular embolism, resulting in a heavy burden on healthcare systems^2^. In China, cardioembolic stroke from AF accounts for 8~13% of all ischemic strokes and results in greater disability than other causes of stroke^3^,^4^. Moreover, a one-point increase in the CHA_2_DS_2_-VASc score is associated with an approximate 1.1 to 1.3-fold increase in the risk of poor outcomes^5^.

In 2011, the failure of aspirin to reduce stroke in AF was emphasized to ensure the appropriate use of oral anticoagulants^6^. Oral anticoagulants have been shown to significantly reduce the frequency and severity of strokes among AF patients^7^, and anticoagulant drugs were again confirmed to be effective by the 2014 American College of Cardiology/American Heart Association (ACC/AHA) guidelines. It is critical to determine whether the eligible individuals with AF receive the appropriate antithrombotic management.

Several recent studies^8^,^9^,^10^ have demonstrated that race- or ethnicity-dependent disparities exist in the incidence, symptoms, comorbidity burdens and outcomes of many cardiac conditions, including heart failure (HF) and AF. In addition, among patients with HF and AF, minority racial groups were shown to underutilize treatment and have disproportionately higher risks of inpatient death^11^. In regards to treatment strategies, studies have shown that ethnicity may affect anticoagulation^8^,^10^,^12^ and cardiac ablation^13^ in patients with AF. However, ethnic differences in hospital populations with AF are considered controversial, especially in anticoagulant management^14^,^15^.

The Uygur accounts for approximately 46% of the population in Xinjiang. This ethnic group suffers higher morbidity and mortality from stroke and venous thromboembolism^16^ and has been previously reported to face a disproportionate burden of stroke^16^. Given their unique risk, Uygur may represent a population likely to benefit from anticoagulation therapy. We sought to determine whether hospitalized patients with AF present with significant ethnic differences in terms of clinical features and appropriate therapies.

AF is a leading cause of stroke and that Xinjiang is a typical place to investigate ethnic differences between the Han and Uygur ethnic groups. Thus, we sought to assess the clinical profiles and current management strategies in Han and Uygur patients, to compare these findings to current evidence-based guidelines and recommendations, and to determine whether the influence of ethnicity is observed in the characteristics evaluated in this trial.

## Methods

### Participants and study design

This retrospective study accumulated AF cases from administrative data obtained from 13 hospitals throughout different areas of Xinjiang. According to the Criteria for the Evaluation of General Hospital (2011) published by the Chinese Ministry of Health, Chinese hospitals are classified as Level 1 (community hospitals with only the most basic facilities and very limited inpatient capacity), Level 2 (hospitals with at least 100 inpatient beds providing acute medical care and preventative care services to populations of at least 100,000), and Level 3 (major tertiary referral centers in provincial capitals and major cities, with at least 500 inpatient beds, providing high-level medical services to several geographic regions). There are 15 Level 3 hospitals in Xinjiang, of which 13 were invited to participate (two hospitals were excluded due to refusing to participate). The main purpose of this observational study was to determine the real-world status of anticoagulation in Xinjiang and to identify any differences in ethnicity. This adaptive design also ensured geographic and provider heterogeneity. This study was approved by the Ethics Committee of the First Affiliated Hospital of Xinjiang Medical University (201400422-01) and was performed in accordance with the Declaration of Helsinki. Written informed consent was obtained from all subjects. Patient anonymity was guaranteed and the data were used only for the present study.

Patients information in the data sources was identified by the first 5 discharge diagnosis codes from all hospitalizations for AF between Jul 1, 2014 to Jun 31, 2015. Incident AF events (International Classification of Diseases, Ninth Revision, Clinical Modification, ICD-9-CM: 427.31) were identified using the hospital administrative datasets. Reviews of the medical records showed a 95% positive predictive value of ICD-9 codes in diagnosing AF in this study. Patients in the selected hospitals were included if they were ≥18 years old. The exclusion criteria were as follows: Patients with valvular atrial fibrillation, patients having other diseases with life expectancy <1 year, and patients with atrial flutter.

### Covariate assessment

Previous event-free status and the presence of comorbidities were ascertained through a review of the pre-index admission data. Age, gender, ethnic, BMI, ≤12 years of education and medical insurance were the characteristics acquired from medical histories.

In term of risk assessment for stroke, the CHADS_2_ and CHA_2_DS_2_-VASc stroke risk score were used according to the international guidelines. HAS-BLED scores were used to judge the risk of bleeding. The CHA_2_DS_2_-VASc score was used to assess the appropriateness of anticoagulant therapy according to the international guidelines. As the study was conducted in Jun, 2014, the 2014 guideline was used^17^. Appropriate treatment for patients at low risk for thrombotic complications (CHA_2_DS_2_-VASc = 0) consists of no anticoagulation treatment or aspirin treatment; for intermediate risk patients (CHA_2_DS_2_-VASc = 1), aspirin or oral anticoagulation (OAC) treatment is recommended; and for high risk patients (CHA_2_DS_2_-VASc ≥2), OAC prescription is indicated. In cases of non-treatment or under-treatment, patients with CHA_2_DS_2_-VASc = 1 did not accept aspirin or antithrombotic treatment, and patients with CHA_2_DS_2_-VASc ≥2 took only aspirin or took neither antiplatelet drugs or anticoagulation treatment. In cases of possible overtreatment, CHA_2_DS_2_-VASc = 0 indicated the use of antithrombotic treatment; CHA_2_DS_2_-VASc = 1 indicated the use of both aspirin and an oral anticoagulant; and CHA_2_DS_2_-VASc ≥2 indicated the use of both aspirin and an oral anticoagulant or the use of more than one type of oral anticoagulant. The judgment regarding overtreatment also considered comorbidities, such as AF complicating acute coronary syndrome (ACS), according to the guidelines.

Clinical data were obtained for each patient to allow for the calculation of the aforementioned stroke risk scores for each individual. Antiplatelet and anticoagulant prescriptions were also identified, and individual medical records were reviewed for any possible contraindications to anticoagulation therapy.

### Statistical analysis

Baseline clinical characteristics, including demographics, medical history, type of AF, procedure and medical therapies, were compared among Han and Uygur patients. Categorical variables are presented as frequencies and percentages. Continuous variables are reported as median ± standard deviation (SD) as appropriate. Differences between the groups were assessed using chi-squared tests and two-tailed *t*-tests. A multivariable logistic regression analysis was used to identify the odds of being at high risk of under-anticoagulation management. All models were adjusted for variables with potential influence on the application of anticoagulation management, including age, gender, education level, hospital type and comorbidities. In both analyses, the standard errors were adjusted to account for the effect of clustering at the practice level. All P values were based on 2-sided *t*-tests and chi-squared tests, and P < 0.05 was used to establish the significance of the tests. All analyses were performed using SPSS V.17.0.

## Results

### Patients characteristics

**Figure 1:**
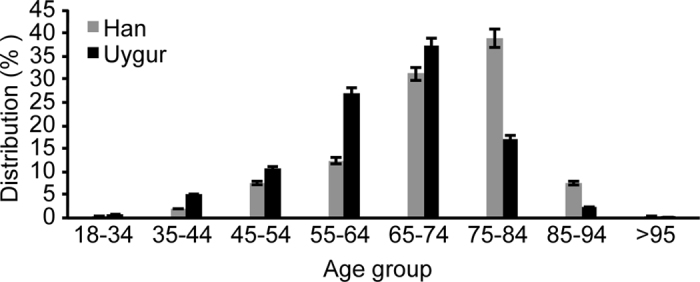
Age distribution of different ethnicity patients with AF.

A total of 4181 cases with AF were identified at 13 sites from Jul 1, 2014 to Jun 31, 2015. 2990 (71.5%) patients were Han and 1191 (28.5%) were Uygur, as shown in Table 1. Compared with Han participants, Uygur participants were younger; had higher body mass index (BMI), and a higher prevalence of HF with reduced ejection fraction and chronic obstructive pulmonary disease (COPD). Han participants presented with higher incidents of hypertension, diabetes mellitus (DM) and stroke. The distribution of AF prevalence showed that the prevalence increased with age; the prevalence of AF was greatest in Han individuals between 75~85 years old and Uygur individuals between 55~74 years old (Fig. 1).

### AF characteristics

In terms of risk assessment, Han patients had higher stroke risk and bleed risk. In contrast, mean CHADS_2_ scores (3.2 ± 1.9 vs. 2.5 ± 1.8, P < 0.001), CHA_2_DS_2_-VASc scores (3.4 ± 1.8 vs. 2.9 ± 1.8, P < 0.001) and HAS-BLED scores (1.9 ± 0.6 vs. 0.6 ± 0.7, P < 0.001) were higher in Hans compared to Uygur. Han patients were at high risk of stroke (CHA_2_DS_2_-VASc score ≥2, 80.6% vs 73.7%, P < 0.001) and high hemorrhage risk (HAS-BLED score ≥3, 14.3% vs 9.2%, P < 0.001) (Table 2). Only 15 patients (0.3%) in this investigation presented with a history of hemorrhagic stroke. Of the patients with AF, 158 (5.3%) Han patients and 36 (3.0%) Uygur patients had ACS.

### AF management strategy

Overall, 37.1% of Han patients and 23.6% of Uygur patients were managed with the rate control strategy (P < 0.001), while 3.5% of Han patients and 1.6% of Uygur patients were managed with rhythm control therapy (P < 0.001). Consistent with the rate- and rhythm-controlled strategies, Han patients underwent more prior cardioversions and prior interventional therapy for AF.

Concerning treatments with anticoagulants, approximately 71.2% patients were undertreated, 8.7% were over-treated, and only 20.0% were appropriately treated. There were no significant differences between undertreatment and appropriate treatment in either the Han or Uygur patients (71.1% vs. 71.3%, P = 0.940; 21.1% vs. 17.5%, P = 0.008). Overused bias existed in both groups (Han 7.8% vs. Uygur 11.3%, P < 0.001) (Table 3). A high proportion of patients with AF (1885, 45.1%) took an antiplatelet drug such as aspirin; of these patients, 1259 (42.1%) were Han and 626 (52.6%) were Uygur. High proportions of patients (45.2%) with high risk patients (CHA_2_DS_2_-VASc ≥2) were using aspirin as the only measure for thromboprophylaxis, 42.1% in Han and 54.8% in Uygur.

The multivariate analyses showed no clear differences in the odds of undertreatment due to lower prescriptions either by ethnic group (odds ratio [OR] 0.83, 95% confidence interval [CI] 0.62–1.11). However, age >65 years old, female gender, education <12 years, admission to a nonteaching hospital, accompanying hypertension, CHD and diabetes were significant predictors of underutilization (Table 4).

## Discussion

### Main findings

In this study, we found that most patients with AF in Xinjiang in northwest China were at high risk of stroke. Only 20.0% were strictly treated in conformance with the anticoagulation guidelines. A conflicting phenomenon exists in older patients with AF: these patients have a high risk of stroke but are treated with insufficient amounts of anticoagulants. The age distribution indicated that AF may occur earlier than expected in Uygur individuals, suggesting that the outcome may be more serious in Uygur patients. Ethnic differences existed in the age distribution, rate control, rhythm control and overtreatment with anticoagulants. Given that all of these factors may have peculiar characteristics that significantly influence the outcome of AF, knowledge of these determinants may help to improve the quality of care for patients of different ethnicities with AF. There were no significant ethnic differences in terms of undertreatment or appropriate treatment with anticoagulants.

### AF, clinical characteristics and ethnicity

As expected, the prevalence of AF in our study sharply increased with age and was most prevalent at the mean age of 69.5 years old. The age-stratified prevalence was higher than that of the general population at 32.9% for ages 65～74 years (compared with 5% in community-based studies^18^), especially in the Han ethnic group. This finding indicates that hospitalized patients are of a higher average age than the general population^19^,^20^.

Hypertension, diabetes and stroke were more prevalent among Han patients, which may have occurred partly because age-related changes in physiology and metabolism affect all organ systems. The responses of the elderly often differing from those of a younger counterpart, and hypertension and diabetes are associated with atherosclerotic disease. Moreover, there is less optimism about the application of drugs in elderly patients. These factors likely contributed to the higher stroke comorbidity in Han patients.

In our cohort, Uygur patients were 6 to 7 years younger than Han patients, suggesting that the age of onset of AF in Uygur individuals may occur earlier than expected^1^. Patients with AF have an age-adjusted risk of stroke that is five-fold higher compared to the normal population regardless of the type of AF^21^. If the ventricular rate is uncontrolled for a prolonged period, tachycardia-mediated cardiomyopathy can occur^22^. Other comorbidities, such as hypertension, heart failure, diabetes and COPD, occur as age increases^23^. Our analysis provided estimates that the future outcome of AF may be worse in Uygur patients than in Han patients.

The reasons for the observed ethnic differences in the clinical characteristics of AF patients are not well understood. Genetic susceptibility has become an increasingly recognized hypothesis to explain racial differences observed in patients with AF^24^. This may be especially true in younger populations^25^. The relative strength of race and ethnicity compared with other established AF risk factors^25^ suggests the mechanism responsible for the racial or ethnic association with AF and the important role that ethnicity plays in disease pathogenesis. However, more evidence is still needed. Determining the direction of this relationship is critical to identifying the causal mechanism responsible for this association, which could be broadly applicable to all patients with or at risk for AF.

### AF and therapy management

Stroke is a massive health issue in China, with 1.8 million deaths from stroke each year; this figure amounts to nearly a third of all stroke deaths worldwide^26^. The mortality and dependency rates were significantly higher in the AF group than in the non-AF group after stroke^27^. Anticoagulation has a definite and significant net clinical benefit. However, our data demonstrate that almost 80% patients with AF in Xinjiang did not receive anticoagulant therapy. Even when approximately 60% of hospitalized patients accepted anticoagulation therapy, only 25% persisted through follow-up^28^. From this perspective, the real status of anticoagulation is rather discouraging. Previously, we found a similar phenomenon^11^ in which a cardioversion and catheter ablation were significantly underutilized in minority ethnic groups.

Conversely, stroke risk scores can also identify subjects who are likely to be at low risk of stroke. The patients with CHA_2_DS_2_-VASc score = 0 who receive anticoagulants may incur bleeding hazards that outweigh any reductions in thromboembolism. Approximately 10% of patients in this study were overtreated; this approach is neither safe nor effective for stroke prevention in patients with AF.

Many studies have demonstrated the gap between the real world and guideline recommendations. The rate of treatment with anticoagulants in hospitalized patients with AF changes over wide range (43.0 to 88.1%)^10^,^19^,^28^. Our study shows that the undertreatment of AF patients is higher than in previous reports. Some reasons may explain the low usage of anticoagulants. First, there was 7-fold difference of the rate of application of anticoagulants between the nonteaching hospitals and teaching hospitals, and physician choices and knowledge regarding the use of anticoagulants in AF may differ significantly from the guidelines^29^. Second, previous studies have suggested that older patients receive fewer guideline-recommended diagnostic studies and treatments compared with younger patients^15^,^30^. Our study confirms this finding. One possible explanation may be the concern about a higher risk of hemorrhage in elderly patients. These results imply that aging-associated diseases and the loss of therapy are associated with functional impairment and disability. Finally, numerous reports have shown that differences in education and knowledge can affect the application of anticoagulants^31^,^32^. Our findings expand on the existing literature describing differences between Han and Uygur populations. Accepting an education of less than 12 years may play a role in determining any patient’s attitudes, knowledge and adherence toward oral anticoagulants^32^,^33^,^34^. Moreover, Uygur individuals face language barriers and live specific lifestyles that likely result in conservative views towards health conditions. Our findings suggest that it is crucial to highlight the treatment of elderly patients with AF and identify the undertreated patients to reduce poor outcomes among the patients with AF in China.

Our study confirms previous observations that more patients are undertreated than overtreated. The reasons for overtreatment with anticoagulants include comorbidity with ACS, the acceptance of percutaneous coronary intervention or endoprostheses, the incidence of pulmonary embolism, and other conditions. However, the reasons for overtreatment are unclear in 13% of overtreated patients.

### Limitations

Although we consider our conclusions to be valid, our study has some limitations. First, it must be noted that this study is limited by its dependence on retrospective databases that include a locally, but not nationally, representative sample. Thus, the sampling was subject to certain judgment biases. We do not have specific data regarding other risk factors, method of anticoagulation, compliance and other laboratory data, which may be important confounding factors. Second, all of the patients were lost to follow-up. The main dangers caused by AF are complications and the failure to appropriately treat patients from different ethnic groups is troublesome, given the risk for poor outcomes among these populations. The differential effects in terms of efficiency and adverse effects based on ethnicity are still unknown. Our findings highlight the need for additional studies to assess the safety, efficacy and outcomes of anticoagulant management in ethnic populations. Third, we do not have sufficient data to ascertain whether there were between-group differences in terms of the subtype of stroke (embolic vs nonembolic). This question will be addressed in a future study. Fourth, ICD-9 codes were used to identify AF and comorbidities in this study. A previous study^35^ found that ICD-9 coding for AF and other stroke risk factors is 80% sensitive and specific. Finally, our cohort consisted of patients with previous AF hospitalization and may not be representative of all AF patients.

This study suggests that the under-prescription of anticoagulants remains associated with increased risk in all ethnic groups, particularly at older ages. Practices and commissioning organizations should adopt optimal management practices to change this situation. Further studies are needed to investigate the influence of ethnic differences on outcomes of real-world anticoagulation treatments.

## Additional Information

**How to cite this article:** Cheng, X. *et al*. Ethnicity and anticoagulation management of hospitalized patients with atrial fibrillation in northwest China. *Sci. Rep.* **7**, 45884; doi: 10.1038/srep45884 (2017).

**Publisher's note:** Springer Nature remains neutral with regard to jurisdictional claims in published maps and institutional affiliations.

## Figures and Tables

**Table 1:** Baseline characteristics of hospitalized patients classified by ethnicity.

Demographics	Overall N = 4181	Han N = 2990	Uygur N = 1191	P
Age (years) (mean ± SD)	69.5 ± 11.9	71.3 ± 11.3	64.9 ± 11.3	<0.001
Female gender (%)	1763 (42.2)	1270 (42.5)	493 (41.4)	0.53
BMI (kg/m^2^) (mean ± SD)	25.9 ± 13.2	25.3 ± 12.1	27.0 ± 13.6	0.03
Type of AF				<0.001
First diagnosed AF (%)	132 (3.2)	76 (2.5)	56 (4.7)	
Paroxysmal AF (%)	1177 (28.2)	928 (31.0)	249 (20.9)	
Persistent AF (%)	805 (19.3)	652 (21.8)	153 (12.9)	
Permanent AF (%)	2066 (49.4)	1334 (44.6)	732 (61.5)	
Health insurance coverage (%)	3717 (88.9)	2641 (88.3)	1074 (90.2)	0.086

**Table 2:** Stroke and bleeding risk factors and other comorbidities.

Stroke risk factors	Overall N = 4181	Han N = 2990	Uygur N = 1191	P
LV EF ≤40% (%)	185 (4.4)	117 (3.9)	68 (5.7)	<0.001
Hypertension (%)	469 (11.2)	360 (12.0)	109 (9.2)	0.01
Age ≥75 (%)	1805 (43.2)	1444 (48.3)	361 (30.3)	<0.001
Diabetes mellitus (%)	844 (20.2)	629 (21.0)	215 (18.1)	0.03
Stroke/TIA (%)	583 (13.9)	470 (15.7)	113 (9.5)	<0.001
Age 65～74 (%)	1369 (32.7)	913 (30.5)	456 (38.3)	<0.001
Female (%)	1763 (42.2)	1270 (42.5)	493 (41.4)	0.53
CHADS_2_ score (mean, ±SD)	2.9 ± 1.9	3.2 ± 1.9	2.5 ± 1.8	<0.001
CHA_2_DS_2_-VASc score (mean ± SD)	3.2 ± 1.6	3.4 ± 1.8	2.9 ± 1.3	<0.001
CHA_2_DS_2_-VASc score ≥2	3289 (78.7)	2411 (80.6)	878 (73.7)	<0.001
0 (%)	485 (11.6)	326 (10.9)	159 (13.4)	
1 (%)	407 (9.7)	253 (8.5)	154 (12.9)	
2 (%)	487 (11.6)	328 (11.0)	159 (13.4)	
3 (%)	629 (15.0)	422 (14.1)	207 (17.4)	
4 (%)	991 (23.7)	709 (23.7)	282 (23.7)	
5 (%)	879 (21.0)	697 (23.3)	182 (15.3)	
6 (%)	263 (6.3)	222 (7.4)	41 (3.4)	
>7 (%)	40 (1.0)	33 (1.1)	7 (0.6)	
CHA_2_DS_2_-VASc score ≥2	3289 (78.7)	2411 (80.6)	878 (73.7)	<0.001
Bleeding risk factors				
HAS-BLED score (mean ± SD)	1.4 ± 0.9	1.90 ± 0.55	0.6 ± 0.66	<0.001
0 (%)	910 (21.8)	585 (19.6)	325 (27.3)	
1 (%)	1102 (26.4)	805 (26.9)	297 (24.9)	
2 (%)	1633 (39.1)	1173 (39.2)	460 (38.6)	
3 (%)	497 (11.9)	395 (13.2)	102 (8.6)	
4 (%)	31 (0.7)	24 (0.8)	7 (0.6)	
5 (%)	6 (0.1)	6 (0.0)	0 (0.0)	
≥6 (%)	2 (0.0)	2 (0.0)	0 (0.0)	
HAS-BLED score ≥3	536 (12.8)	427 (14.3)	109 (9.2)	<0.001
Other co-morbidities				
History of CHD	1579 (37.8)	1136 (38.3)	443 (37.2)	0.53
Myopathy	216 (5.2)	144 (5.0)	72 (6.1)	0.22
COPD (%)	85 (2.0)	42 (1.4)	43 (3.6)	<0.001
History of thyroid disease (%)	497 (11.9)	374 (12.5)	123 (10.3)	0.05

HF, heart failure; CHD, coronary artery heart disease; COPD, chronic obstructive pulmonary disease.

**Table 3:** Management strategy in hospitalized patients with AF.

AF management strategy	Overall N = 4181 (%)	Han N = 2990	Uygur N = 1191	P
Rate control	1388 (33.2)	1107 (37.1)	281 (23.6)	<0.01
Rhythm control	124 (3.0)	104 (3.5)	20 (1.7)	<0.01
Prior cardioversions	308 (7.4)	278 (9.3)	30 (2.5)	<0.001
Prior interventional therapy for AF	129 (3.1)	114 (3.8)	15 (1.3)	<0.001
CHA_2_DS_2_-VASc-score and anticoagulation strategy*				
0	347 (8.3)	N = 260	N = 87	
A		230 (7.7)	67 (5.6)	0.02
O		30 (1.0)	20 (1.7)	0.08
1	592 (14.2)	N = 365	N = 227	
U		109 (3.6)	67 (5.6)	0.01
A		126 (4.2)	80 (6.7)	0.01
O		130 (4.3)	80 (6.7)	0.01
≥2	3242 (77.54)	N = 2365	N = 877	
U		2017 (67.5)	782 (65.6)	0.28
A		275 (9.2)	61 (5.1)	<0.001
O		73 (2.4)	34 (2.9)	0.45

*A, appropriate treatment; O, possible overtreatment; U, absence of treatment or under-treatment.

**Table 4:** Multivariable analysis of factors associated with undertreated anticoagulation in patients with AF.

Variables	Adjusted OR	95% CI	P
Age >65	14.83	11.44 to 19.24	<0.001
Education <12 years	1.82	1.63 to 2.06	0.03
Nonteaching hospital	7.53	5.67 to 9.23	<0.001
Han	0.83	0.62 to 1.11	0.22
Hypertension	2.38	1.65 to 3.44	<0.001
CHD	1.33	1.05 to 1.68	0.02
Diabetes	1.76	1.30 to 2.37	<0.001
COPD	1.65	0.56 to 4.87	0.37
Stroke	1.01	0.71 to 1.44	0.95

HF, heart failure; CHD, coronary artery heart disease; COPD, chronic obstructive pulmonary disease.
